# NS1643 enhances the activity of T1019PfsX38 and Q1070X hERG channels associated with long QT syndrome type 2

**DOI:** 10.1007/s00424-026-03215-7

**Published:** 2026-10-02

**Authors:** Majid K. Al Salmani, Amira Al Kharusi, Mohammed Al Rawahi, Sulayma Albarwani

**Affiliations:** 1 Department of Physiology, College of Medicine and Health Sciences, Sultan Qaboos University, Muscat, Oman; 2 Sultan Qaboos University Hospital, Muscat, Oman

**Keywords:** HERG, KCNH2, K_V_11.1, Long QT syndrome, Cardiac arrhythmia

## Abstract

Type 2 long QT syndrome (LQT2) increases the risk of cardiac arrest and sudden death due to reduced rapid delayed rectifier potassium currents (IKr) in the heart. Caused by loss-of-function genetic variants in the hERG K^+^ channel, LQT2 might benefit from variant-targeted therapy approaches. T1019PfsX38 and Q1070X are LQT2-causing variants reported in Oman and Saudi Arabia. Although nonsense-mediated mRNA decay (NMD) has been proposed to contribute to reduced expression of these truncating variants, it remains unclear whether residual mutant hERG channel function can be pharmacologically enhanced. We used the patch-clamp technique and examined the effects of the hERG potentiator NS1643 on wild-type-, T1019PfsX38- and Q1070X-hERG channels expressed in HEK293 cells. We started by assessing the response of these variants under the influence of a ventricular action potential clamp protocol, then used additional protocols to elucidate the drug’s mechanism of action. NS1643 [10 µM] enhanced the repolarization currents in wild-type-, T1019PfsX38- and Q1070X-expressing cells. Its effect was associated with accelerated activation rates and a negative shift in the voltage dependence of activation. In addition, NS1643 suppressed fast deactivation transitions. We conclude that the functions of T1019PfsX38- and Q1070X-hERG channels can be augmented by hERG potentiators if NMD is incomplete, which may facilitate the development of a mechanism-based rescue approach.

**Supplementary Information:**

The online version contains supplementary material available at https://doi.org/10.1007/s00424-026-03215-7.

## Introduction

The human Ether-à-go-go-Related Gene (hERG, K_V_11.1, *KCNH2*) encodes a voltage-dependent K^+^ channel highly expressed in cardiac myocytes and involved in regulating their electrical activity. It mediates the efflux of K^+^ during the repolarisation phase of the cardiac action potential (AP) and, thus, controls the length of time between the initiation of ventricular depolarisation and the end of ventricular repolarisation [1]. This duration corresponds to the time between the beginning of the Q wave and the end of the T wave (QT interval) of the electrocardiogram (ECG). Loss-of-function genetic variants in hERG prolong the QT interval and cause long QT syndrome (LQTS) type 2 (LQT2) [2]. In contrast, gain-of-function mutations in hERG cause short QT syndrome type 1 (SQT1) [3, 4]. LQT2 is more common than SQT1, and both conditions have deleterious cardiac health consequences [5]. Clinically, affected patients may present with palpitations and fainting spells, but the first presentation in most cases is cardiac arrest or sudden death [6].

Many compounds can block hERG but only a few can increase its activity. Depending on the context, pharmacological targeting can be two-edged. Blockers of hERG cause acquired LQTS and many preclinical drugs are excluded from further development due to this risk [7]. Despite that, hERG blockers such as dofetilide have antiarrhythmic effects and are used primarily in the treatment of atrial fibrillation [1, 8]. Unlike blockers, no drugs are currently in clinical use that work by enhancing hERG channel activity [9]. Such drugs, termed activators or potentiators, could theoretically modulate the QT interval duration by increasing the activity of hERG during repolarisation. This approach can potentially treat certain types of LQTS. The closest application of such approach would be patients with LQT2 who carry hERG dysfunction but retain partial or full channel expression. The applicability of such drugs is limited by complex hERG channel function and the variety of molecular pathologies caused by LQT2 mutations. In addition, excess repolarisation reserve caused by hERG potentiator use can be proarrhythmic if the dosage is not properly adjusted [10].

LQT2 variants cause different molecular pathologies and hERG potentiators work through multiple mechanisms. hERG variants can impair protein synthesis (class I); folding, assembly and trafficking (class II); channel gating (class III); and/or K^+^ flow through the pore (class IV) [11, 12]. Potentiators of hERG may slow the channel deactivation process (type I), shift the voltage dependence of inactivation to the right (type II), shift the voltage dependence of activation to the left (type III), and/or increase the open probability of the channel (type IV) [9, 13]. While hERG potentiators are particularly effective in correcting class III defects, they may also enhance the function of variants from other classes that exhibit residual cell-surface expression.

Amongst > 1000 known hERG channel variants, a number have been functionally characterised and a fraction of those have been pharmacologically targeted to test their responses to available investigational drugs. Missense mutations constitute about 60% of the reported LQT2 variants [14] and were a subject of few large-scale studies. Anderson et al. [15] studied about 170 missense mutations using Western blotting and found that the majority of LQT2 missense mutations invoke Class II trafficking defects. The expression of 64% of these variants could be experimentally corrected using either low temperature incubation or E-4031 corrector action. Mutations at the pore region were more resistant to rescuing by low temperature and E-4031 treatment when compared to the rest of the protein. In a more recent study, O’Neill et al. [16] used the automated patch clamp technique to measure the electrophysiological function of 533 different KCNH2 missense variants at a heterozygous state. By directly recording current densities, they provided data that refined variant classification and improved long-term modelling of cardiac event outcomes.

Non-missense variants, such as frameshift and nonsense mutations, constitute about 40% of the LQT2 causing variants [14]. Many of them are believed to be recognised and eliminated at the mRNA level by nonsense-mediated mRNA decay (NMD) (Gong et al. 2014). Many of these truncated channels have normal channel function if they reach the plasma membrane. Thus, NMD in this case worsens the disease by destroying potentially functional hERG channels. However, the efficiency of the NMD process is not uniform in humans. Inter-individual variabilities affect how efficient the NMD process is, a fact that should be considered when testing and making decisions about mutation-specific counselling and therapeutic interventions [17]. In addition, NMD can be modulated pharmacologically to reduce its unwanted effects.

In this article we aim at characterising the effects of the hERG potentiator NS1643 on wild-type hERG and two LQT2 genetic variants reported in the Arabian Peninsula. T1019PfsX38- and Q1070X-hERG were reported in LQT2 families in Oman and Saudi Arabia, respectively [18–20]. Children who are homozygous for T1019PfsX38- and Q1070X-hERG showed a very severe clinical phenotype that starts even before birth. T1019PfsX38 was found in a large family and was first noted in a homozygous 5-year-old girl with bradycardia, intermittent AV block, paroxysmal VT and a history of foetal tachyarrhythmia [19]. T1019PfsX38 has also been recently identified in a 13-year-old homozygous LQT2 male who had experienced syncope at rest [18]. On the other hand, the Q1070X variant was first identified in a family that suffered repeated early miscarriage. The Q1070X hERG variant was believed to be a direct cause of stillbirth incidences due to debilitating arrhythmias [20]. The pronounced disease severity in these reports necessitates a thorough investigation into the root-cause of the disease, which could lead to the development of potential therapeutic interventions. Little information is available about the functional characteristics of these two LQT2 variants. Structurally, both variants affect the c-tail of hERG but introduce different types of modifications. T1019PfsX38 replaces the last 140 amino acids of hERG with 37 unique amino acids, while Q1070X deletes the last 90 amino acids. The expression of Q1070X-hERG is reduced in the cell due to the activity of the NMD machinery [20]. Inhibition of the NMD increases Q1070X-hERG protein expression in the cells, providing prospects of mechanism-based therapeutic interventions [21, 22].

Electrophysiologically, both variants can produce functional conductive K^+^ channels if they escape the NMD machinery and reach the cell surface. When expressed as cDNA in a heterologous system, Q1070X-hERG exhibits normal voltage dependence of activation but displays faster inactivation and slower recovery from inactivation compared with WT-hERG [20]. Similarly, the T1019PfsX38-hERG variant demonstrates normal voltage dependence of activation and recovery kinetics; however, it shows accelerated inactivation at depolarizing potentials and faster deactivation at hyperpolarizing potentials [23]. When examined using the step-ramp voltage-clamp protocol, T1019PfsX38-hERG produces reduced outward currents relative to WT-hERG, attributable to a negative shift in the voltage dependence of inactivation [23]. Together, the findings from Bhuiyan et al. [20] and Al Salmani et al. [23] indicate that structural alterations introduced by the Q1070X and T1019PfsX38 variants impair channel gating by modifying inactivation and deactivation properties. Whether these pathological effects can be reversed through pharmacological modulation remains unknown.

This study investigates mutation-specific drug responses as a basis for developing targeted therapies. We used an action potential (AP) voltage-clamp protocol to assess the responses of wild-type-, T1019PfsX38-, and Q1070X-hERG channels to the pharmacological potentiator NS1643. NS1643 has been shown to act as a hERG channel activator through multiple mechanisms, including reduced inactivation, slowed deactivation, and altered activation kinetics [24–26]. On this basis, we hypothesized that NS1643 could counteract the gating impairments associated with the T1019PfsX38- and Q1070X-hERG variants at the cell surface. We examined the effects of NS1643 on repolarizing currents carried by these channels when expressed in HEK293 cells and further characterized its impact on hERG channel activation and deactivation. The experiments described below provide mechanistic insight into NS1643 action and evaluate its capacity to partially suppress LQT2-associated dysfunction caused by the T1019PfsX38 and Q1070X hERG variants.

## Materials and methods

### Site-directed mutagenesis

We modified the pcDNA3.0 plasmid that contains WT-hERG cDNA using site-directed mutagenesis as described in Al Salmani et al. [23]. The mutagenesis polymerase chain reaction (PCR) primers for the T1019PfsX38 mutation were 5′-gccccgccccacccccagcc-3′ and 5′-ggctgggggtggggcggggc-3′, and for the Q1070X mutation were 5’-cagctgctacagaggtagatgacgctggtcc-3’ and 5’-ggaccagcgtcatctacctctgtagcagctg-3’ (Eurofins Genomics, Luxembourg). The resulting mutagenesis was confirmed using Sanger sequencing (Eurofins Genomics, Cologne, Germany).

### Mammalian cell culture and transfection

For patch clamp recordings, we used human embryonic kidney 293 (HEK-293) cell line (passage < 40) (ECACC, MERCK, Catalog # 85120602) grown in Dulbecco’s Modified Eagle Medium (DMEM) high glucose (Sigma-Aldrich, St. Louis, MO, USA). Cells were maintained in culture at 37 °C (5% CO_2_) and passaged as described in Al Salmani et al. [23]. Before patch clamp experiments, the cells were seeded onto 35 mm petri dishes (Carl Roth, Karlsruhe, Germany) containing 22 mm glass coverslips (Carl Roth, Karlsruhe, Germany). We transfected the cells 24 h after seeding using Lipofectamine 2000 kit reagents (Thermofisher Scientific, Waltham, MA, USA, Catalog # 11668019) and a mixture of 2.5 µg of hERG plasmid DNA and 1.5 µg of enhanced green fluorescent protein N1 (EGFP-N1) (Clontech, CA, USA, Catalog # 632469). The cells were used for experiments 24–96 h after transfection.

### Electrophysiology solutions, drugs and microelectrode preparation

For electrophysiological recordings, the extracellular solution contained in mM: 140 NaCl, 4 KCl, 2.5 CaCl_2_, 1 MgCl_2_, 10 D-glucose and 5 HEPES, adjusted to pH 7.45 with NaOH. The intracellular solution contained in mM: 130 KCl, 5 MgCl_2_, 5 EGTA, 5 Na_2_ATP and 10 HEPES, adjusted to pH 7.2 with KOH. Fresh Na_2_ATP from 200 mM stock was added to the intracellular solution before every experiment and stored on ice, pH was adjusted accordingly. All chemicals used in the solution were from Carl Roth (Karlsruhe, Germany). NS1643 was purchased from Bio-Techne Ltd. (Abingdon, UK, Catalog # 3062) and was prepared as a 30 mM stock in DMSO.

Glass pipettes were made freshly before each experiment using premium thin wall borosilicate capillary glass with filament (model G150TF-4, Warner Instruments, USA). The pipettes were pulled using PC-100 puller (Narishige, Tokyo, Japan) or P-1000 puller (Sutter Instruments, USA). All pipettes had resistance of ~ 3.6 MΩ when put in the bath solution.

### Patch clamp setup, procedures and data acquisition

Patch clamp experiments were carried out as described in Al Salmani et al. [23]. After transferring the cells onto the recording chamber, we approached them using a glass microelectrode filled with the intracellular solution. Successfully transfected cells were identified by EGFP expression. The recorded currents were amplified using Axopatch 200b and acquired using Digidata 1550B and pClamp 11.2/10.7 software (Molecular Devices, San Jose, CA, USA). The holding potential (V_hold_) was − 80 mV except in Fig. 4 where V_hold_ was − 100 mV. The signals were low-pass filtered at 10 kHz and sampled at 20 kHz, except for action potential clamp measurements, the sampling frequencies were either 2–5 kHz as described in the results section. The extracellular solution was added using a syringe and washed out using a vacuum pump. NS1643 was diluted in the extracellular solution right before perfusion. All voltage-clamp protocols are described in the results section. All recordings were acquired at room temperature (23–26 °C).

### Action potential clamp measurements

We used an action potential clamp waveform modified from [27]. The resulting currents were leak subtracted using P/4 leak subtraction protocol as described in [28]. Briefly, four inverted quarter size AP waveforms were recorded before applying four additional full size AP waveforms to the same cell. Leak subtraction was performed offline. hERG channel activity was assessed as area under the curve between 75% and 95% of the AP duration (APD_(75−95)_) as indicated in Figures S1 and S2. To correct for NS1643-induced inhibition of endogenous currents, we measured the average area (I_*integral*_) under the AP-clamp curves obtained from untransfected HEK293 cells before and after treatment with NS1643 (Figure S1 and S2). The average reduction in APD_(75−95)_ I_*integral*_ (in pA.s/pF) following NS1643 [10 µM] were 0.09 ± 0.05 (*n* = 5) and 0.15 ± 0.05 (*n* = 5) for the 400 ms and the 1000 ms AP-clamp waveforms, respectively (Figure S1 and S2). These numbers were used to correct the values of I_*integral*_ obtained from hERG-expressing cells.

### Data analysis, statistics and software

All patch clamp data and analyses were obtained and fitted using either Clampfit 11.2 or SigmaPlot 14.0 (Systat Software Inc, San Jose, CA, USA). We fitted tail currents with a Boltzmann equation $\:I=\frac{I\:max}{1+\left(Vmid-Vm\right)\:/k)}+C$ to determine the half-maximum voltage (V_mid_) and slope factor (k) of activation. The activation and deactivation rates were obtained by standard fit using 1- or 2-component exponential equations as indicated. Statistical analyses were performed using SigmaPlot 14.0 (Systat Software Inc, San Jose, CA, USA). Data was tested for normality using the Shapiro-Wilk test before performing statistical analyses. Any statistical difference was considered significant if *n* ≥ 4 and *p* < 0.05 using two-tailed Student’s t-test. Figures were made using SigmaPlot 14.0 (Systat Software Inc, San Jose, CA, USA).

## Results

### The effects of NS1643 measured using the ventricular AP clamp

**Fig. 1:**
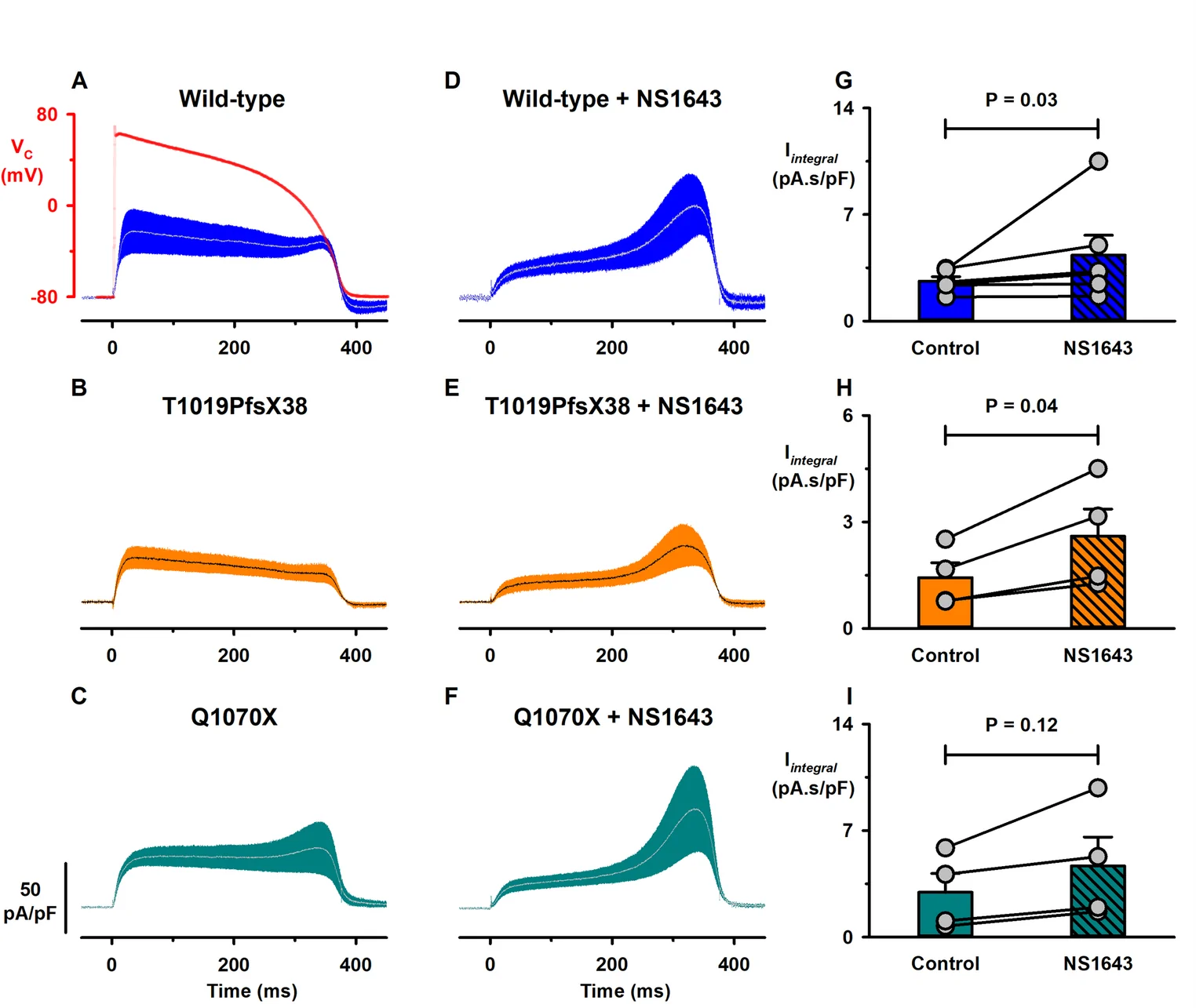
Action potential clamp measurements of the effects of NS1643 on wild-type-, T1019PfsX38- and Q1070X-hERG.** A - F**, Average whole-cell currents from cells expressing different channel variants in the absence and presence of NS1643 [10 µM]. We applied a ventricular AP clamp waveform (red plot in A) and modified the obtained recordings using P/4 leak subtraction protocol. **G-I**, Summary data of the repolarization current integrals (I_*integral*_) measured as the area under the curve between 300 and 380 ms (APD_(75−95)_) of the AP duration. The treatment points in G - I were corrected for NS1643-induced inhibition. V_C_, clamp voltage; *n* = 4–6, paired Student’s t-test. Other details as in the Methods and Results sections and Figure S1

As an initial step, we employed the AP clamp technique to study WT-, T1019PfsX38-, and Q1070X-hERG channel activity in the absence and presence of NS1643 (10 µM). The AP clamp protocol exposes cells to dynamic voltage changes that follow a predefined cardiac action potential waveform, allowing measurement of hERG K^+^ currents under quasi-physiological conditions. Under control conditions, AP clamp currents exhibited two distinct peaks: one immediately after depolarization and another during the repolarization phase (Fig. 1A–C). The latter peak reflected hERG channel activity, as it was absent in recordings from untransfected HEK293 cells (Figure S1). To quantify hERG channel function, we calculated current integrals (I_*integral*_), which capture the combined effects of time- and voltage-dependent channel behaviors. Outward hERG currents peaked between 75% and 95% of the action potential duration (APD_*(75–95)*_), which was defined as the repolarization phase. In untransfected HEK293 cells, the repolarization I_*integral*_ accounted for 5.7 ± 0.6% (*n* = 5) of the total I_*integral*_, whereas in WT-, T1019PfsX38-, and Q1070X-hERG–expressing cells, the corresponding values were 19.5 ± 2.6% (*n* = 6), 15.6 ± 2.3% (*n* = 4), and 18.9 ± 4.4% (*n* = 4), respectively.

Figure 1D–F shows that NS1643 [10 µM] altered the current waveforms evoked by the AP clamp protocol in all cell groups. NS1643 generally reduced total I_*integral*_ values, although the extent of inhibition differed between hERG-expressing and untransfected cells. The inhibitory effect was more pronounced in untransfected cells, in which NS1643 reduced total I_*integral*_ by 50.5 ± 11.5% (*n* = 5; Figure S1). In contrast, the reductions in total I_*integral*_ for WT-, T1019PfsX38-, and Q1070X-hERG–expressing cells were 20.6 ± 10.4% (*n* = 6), 17.7 ± 14.0% (*n* = 4), and 15.4 ± 8.9% (*n* = 4), respectively (data not shown). These findings indicate that NS1643 exerts two principal effects: inhibition of endogenous HEK293 currents and enhancement of hERG channel activity.

To account for the inhibitory component, average repolarization I_*integral*_ values were mathematically corrected for the non-hERG current suppression by NS1643, as described in the Methods and indicated in the legends of Figs. 1 and S3. Using the 400 ms AP waveform, NS1643 increased the repolarization I_*integral*_ of WT-, T1019PfsX38-, and Q1070X-hERG channels by 53 ± 32% (*n* = 6), 80.3 ± 7.1% (*n* = 4), and 78 ± 21.4% (*n* = 4), respectively (Fig. 1G–I). These data demonstrate a substantial potentiating effect of NS1643 on the repolarizing function of all three channel variants.

To further characterize NS1643 effects, we repeated the experiments using a longer AP clamp waveform. A 1000 ms AP induced larger repolarization peak currents in all variants compared with the 400 ms AP (Figure S3 vs. Figure 1). This protocol was used to determine whether NS1643 could further enhance hERG K^+^ currents after prolonged depolarization. Figure S3 shows that only T1019PfsX38-hERG exhibited a significant response to NS1643 when the AP was extended to 1000 ms. Under these conditions, NS1643 increased the repolarization I_*integral*_ in T1019PfsX38-hERG–expressing cells by 27.1 ± 8.7% (*n* = 4). In contrast, NS1643 increased the repolarization I_*integral*_ in WT- and Q1070X-hERG–expressing cells by only 10.8 ± 22.7% and 11.8 ± 17.5%, respectively, changes that did not reach statistical significance.

### Properties of NS1643-sensitive endogenous currents of HEK293

**Fig. 2:**
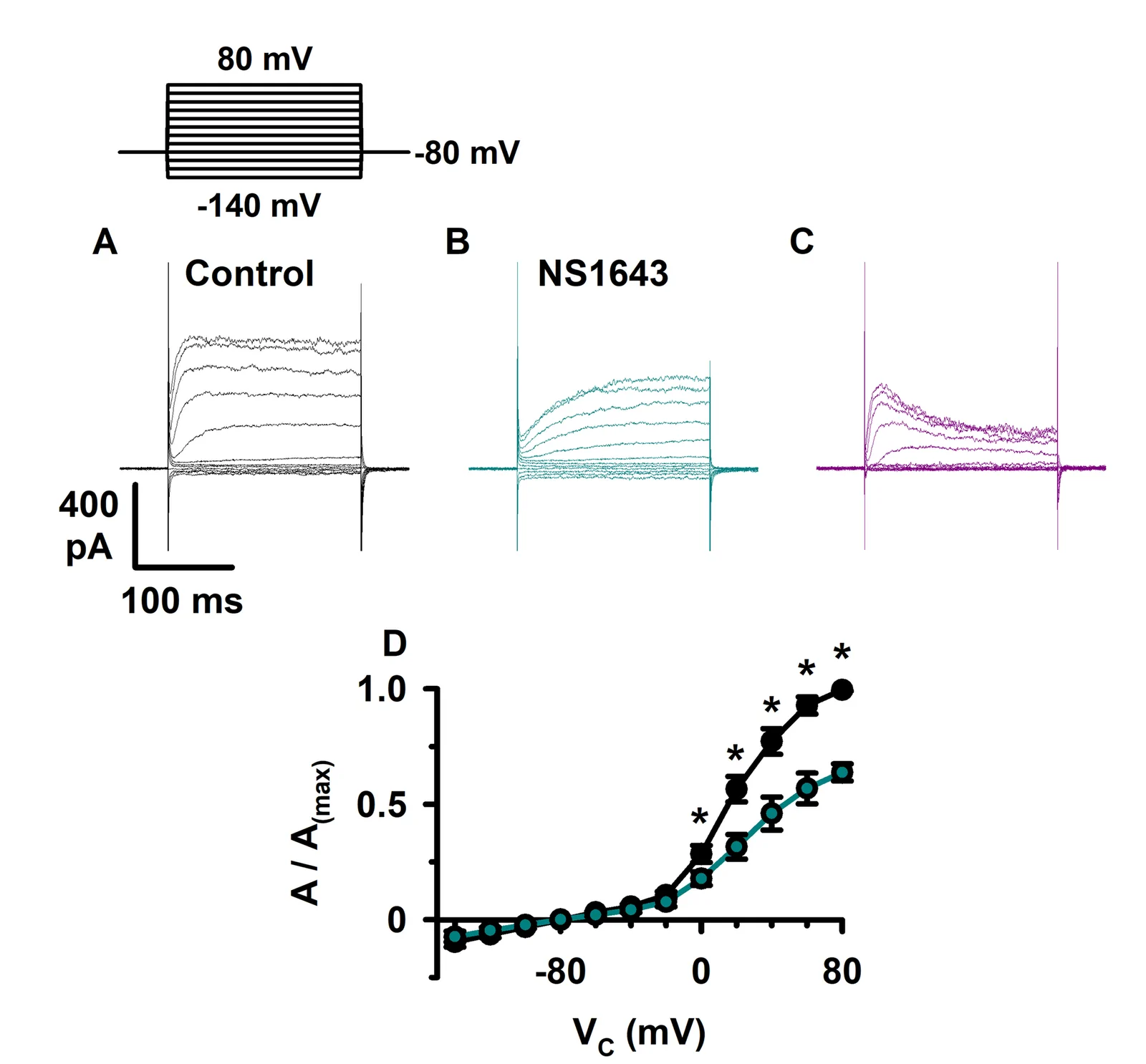
Application of NS1643 to untransfected HEK293 cells.** A**,** B**, Representative currents from HEK293 cells in the absence and presence of NS1643 [10 µM]. The voltage clamp protocol is shown above A. **C**, Currents obtained by subtracting B from A. **(D)** Summary of normalised current integral values (A/A(max)) plotted against the test potential. *n* = 5 each, * *p* < 0.05, paired Student’s t-test

**Fig. 3:**
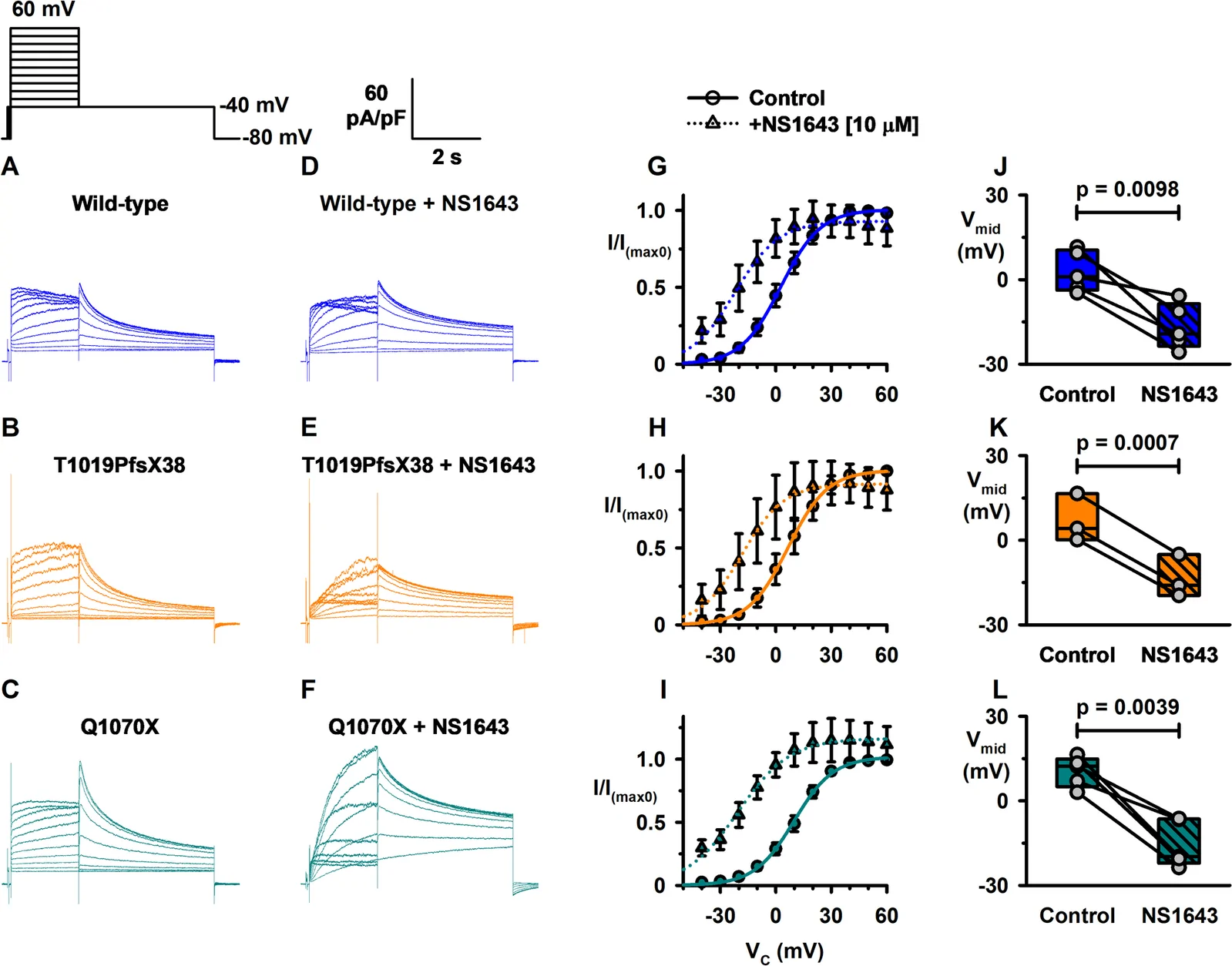
The voltage dependence of activation of wild-type-, T1019PfsX38- and Q1070X-hERG in response to NS1643.** A – F**, Representative currents from HEK293 cells expressing wild-type-, T1019PfsX38- or Q1070X-hERG under control and NS1643 [10 µM] treatment conditions. The voltage clamp protocol is shown above A. **G – I**, Summary data of normalised peak tail currents in response to different test potentials. The baseline zero current was set at the end of the brief 40 ms depolarisation segment to −40 mV before test potentials. Peak currents were normalised by division over maximal peak current before adding the drug (I_max0_). The continuous and dotted lines are best fits to the average data using the Boltzmann equation. **J – L**, Box plots of the half maximum voltage (V_mid_) of activation under control and treatment conditions. *n* = 3–5, other details as in Fig. 1

To our knowledge, no previous study has specifically characterised the effects of NS1643 on endogenous currents in HEK293 cells. Since HEK293 cells are widely used in biomedical research, we sought to define the behaviour of NS1643-sensitive currents under steady-state conditions. Figure 2 shows representative recordings and quantification of currents from untransfected HEK293 cells in response to 200 ms voltage steps from − 140 mV to 80 mV. Figure 2A and B display example current traces recorded in the absence and presence of NS1643, respectively. Visual inspection of these traces reveals an approximately linear current–voltage (I–V) relationship at negative potentials and the appearance of voltage-activated components at more positive potentials. NS1643 reduced both the activation rate and the maximal amplitude of these voltage-activated currents (Fig. 2B). Subtraction of the NS1643 trace (Fig. 2B) from the control trace (Fig. 2A) yielded the NS1643-sensitive current shown in Fig. 3C. These NS1643-sensitive currents activated rapidly, reached their peak within 50 ms, and then inactivated slowly (Fig. 2C).

To quantify the effects of NS1643 on endogenous HEK293 currents, we measured the normalised area (A) under the 200 ms voltage-step currents. Figure 2D shows average A/A_max_ plotted as a function of the corresponding command voltage. Under control conditions, the I–V relationship was linear at voltages below − 20 mV and became sigmoidal at more positive potentials (Fig. 2D). NS1643 reduced the amplitude of the voltage-dependent sigmoidal component at voltages ≥ −20 mV, decreasing the area under the curve by approximately 40% at 80 mV. Overall, NS1643-sensitive endogenous currents in HEK293 cells were voltage-activated and displayed fast activation with slow inactivation at voltages ≥ −20 mV.

### The effects of NS1643 on the voltage dependence of hERG channel activation

hERG is activated by membrane depolarization [29]. The voltage dependence of activation reflects the maximal likelihood that channels transit to the open state at a given membrane potential when sufficient time is allowed to reach steady state. This property is typically assessed by applying prolonged depolarizing steps and measuring tail currents at potentials that permit rapid recovery from inactivation. Tail currents therefore provide a convenient readout of relative activation probability. In this study, the voltage dependence of activation of WT-, Q1070X-, and T1019PfsX38-hERG channels was evaluated under control conditions and during NS1643 exposure. Cells expressing these variants were depolarized from a holding potential of −80 mV to test voltages between − 40 and 60 mV for 2 s in 10 mV increments. Tail currents were then measured at −40 mV (Fig. 3). Figure 3A–C shows representative recordings from WT-, Q1070X-, and T1019PfsX38-hERG under control conditions. This protocol induced characteristic “hooked” tail currents, reflecting rapid recovery from inactivation combined with relatively slow deactivation of hERG at −40 mV. The step currents also contained a component resembling that observed in untransfected HEK293 cells (Fig. 2). Under control conditions, maximal tail current amplitudes did not differ significantly among the three hERG variants.

Following NS1643 application (Fig. 3D–F), three qualitative changes in current waveforms were evident. First, inward rectification of the step currents became more pronounced. Second, maximal tail current amplitudes remained largely unchanged. Third, tail current deactivation appeared slower in the presence of NS1643 than under control conditions. To further characterize activation voltage dependence, peak tail current amplitudes were plotted as a function of test potential. Figures 3G–I summarize average normalized peak tail currents for WT-, Q1070X-, and T1019PfsX38-hERG under control and NS1643 conditions. Under control conditions, the activation curves for the three variants overlapped, indicating no detectable differences in voltage dependence. NS1643 modulated all three variants similarly, shifting the activation curves toward more negative potentials without altering maximal tail current amplitude. To quantify these effects, the activation curves were fitted with Boltzmann functions. Figures 3J–L show V_mid_ values for each variant under control and NS1643 treatment. At baseline, V_mid_ values did not differ significantly between variants (*p* > 0.05) and fell between 0 and 15 mV. The slope factors (k) for WT-, T1019PfsX38-, and Q1070X-hERG under control conditions were 9.8 ± 0.5 (*n* = 5), 9.7 ± 0.2 (*n* = 3), and 9.6 ± 0.7 (*n* = 5), respectively. NS1643 shifted V_mid_ by approximately − 20 mV, yielding average values near − 20 mV for all three variants (Fig. 3J–L). In the presence of NS1643, k values were 7.9 ± 1.1 (*n* = 5), 8.5 ± 1.1 (*n* = 3), and 7.5 ± 0.7 (*n* = 5) for WT-, T1019PfsX38-, and Q1070X-hERG, respectively, with no statistically significant differences between control and treated conditions (*p* > 0.05, paired Student’s t-test). In summary, NS1643 induces an approximately 20 mV negative shift in the voltage dependence of activation without altering the steepness of the activation curve or significantly changing maximal outward current amplitude.

### The effect of NS1643 on the activation kinetics of the hERG variants

**Fig. 4:**
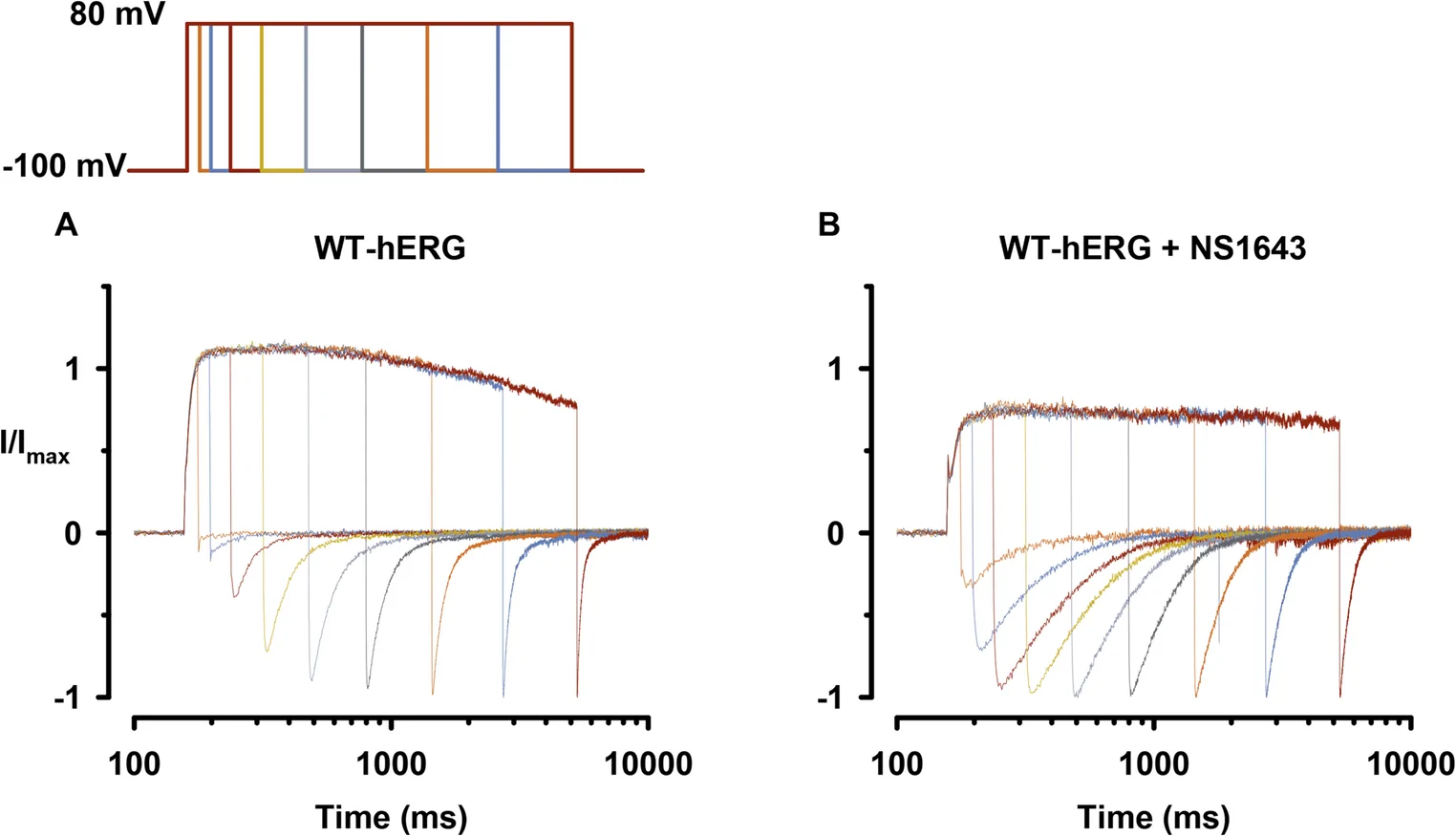
Example recording to assess changes in WT-hERG activation in response to NS1643. **A** and **B**, Representative currents from HEK293 cells expressing wild-type-hERG under control conditions and after the addition of NS1643 [10 µM]. The voltage-clamp protocol is shown above A

**Fig. 5:**
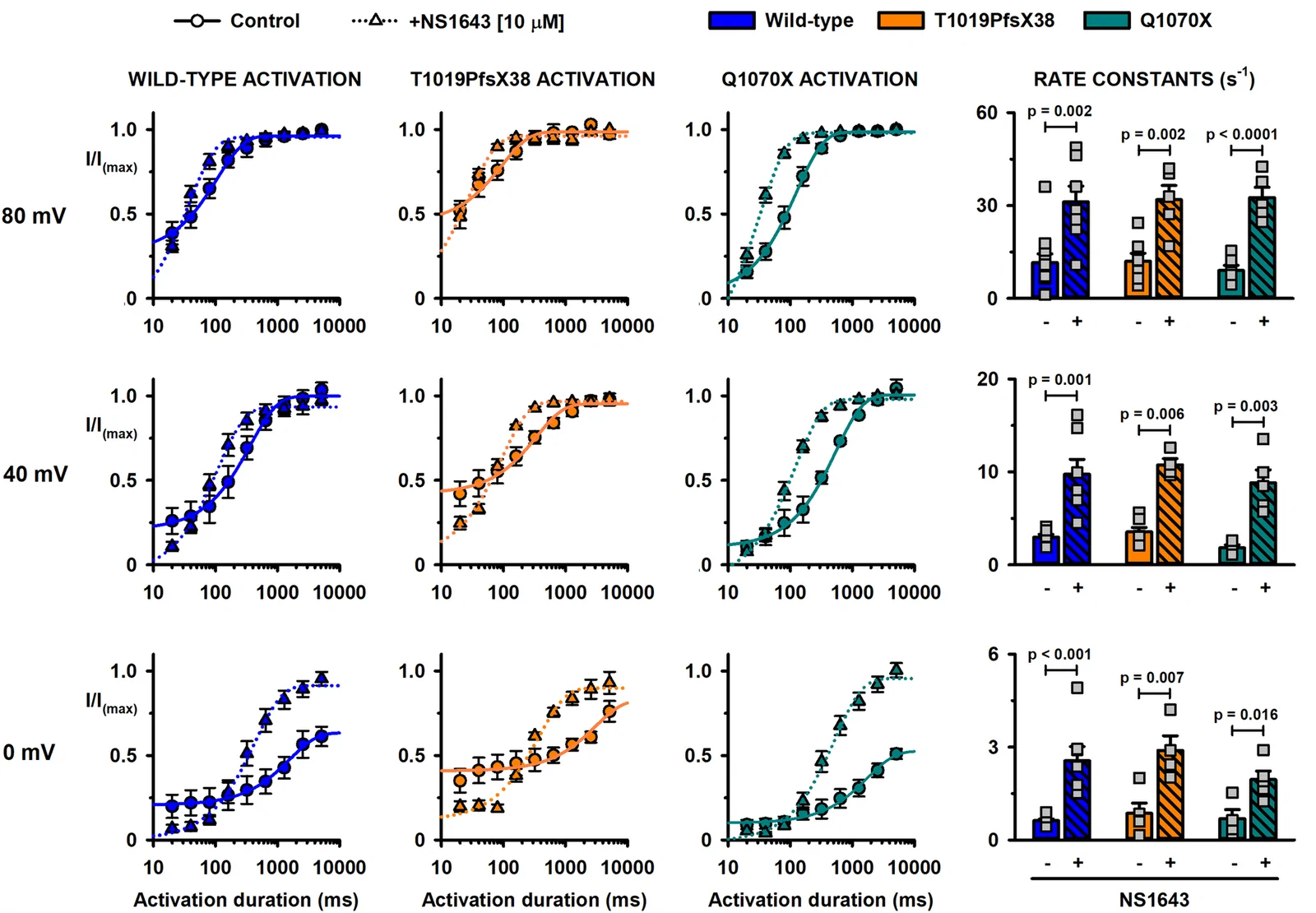
hERG activation kinetic in response to NS1643. Summary data of normalized macroscopic hERG activation extent (I/I_(max)_) plotted against the duration of the activation voltage step (80, 40 or 0 mV) and measured as peak inward tail currents at −100 mV. The continuous and dotted lines are best fits to the average data using exponential equations. I_(max)_ were the maximal tail current values after activation at 80 mV for 5.120 s in the same cell. The bar graphs to the right summarize values of the activation rate constants under control and NS1643 treatment conditions. The voltage clamp protocol follows the same shape as shown in Fig. 1. Wild-type (*n* = 7–12); T1019PfsX38 (*n* = 4–11); Q1070X (*n* = 4–6)

The amplitude of K^+^ currents through hERG channels during the repolarization phase depends on several factors, including the rate of channel activation by voltage. To assess whether NS1643 affects activation kinetics, a voltage clamp protocol was employed to quantify activation rates at different membrane potentials. Specifically, from a holding potential of −100 mV, test pulses of varying magnitudes (0, 40, and 80 mV) and durations (logarithmically spaced from 20 to 5120 ms) were applied, and tail currents were measured at −100 mV. Figure 4 shows representative recording of WT-hERG activity elicited by varying 80 mV activation durations. Figure 5 gathers quantification values of the activation patterns and the activation rate constants at different potentials for the investigated variants. At 0 mV, activation rates for all variants were comparable under control conditions. Activation became progressively faster at higher potentials, with rates at 80 mV more than ten times those at 0 mV across all variants. NS1643 accelerated activation kinetics similarly in all tested channels, with rate constants increasing on average by 2.4-, 2.7-, and 2-fold in the presence of NS1643 at 0, 40, and 80 mV, respectively. Thus, NS1643 consistently accelerates the activation of WT-, T1019PfsX38-, and Q1070X-hERG channels.

### The effect of NS1643 on hERG channel deactivation

**Fig. 6:**
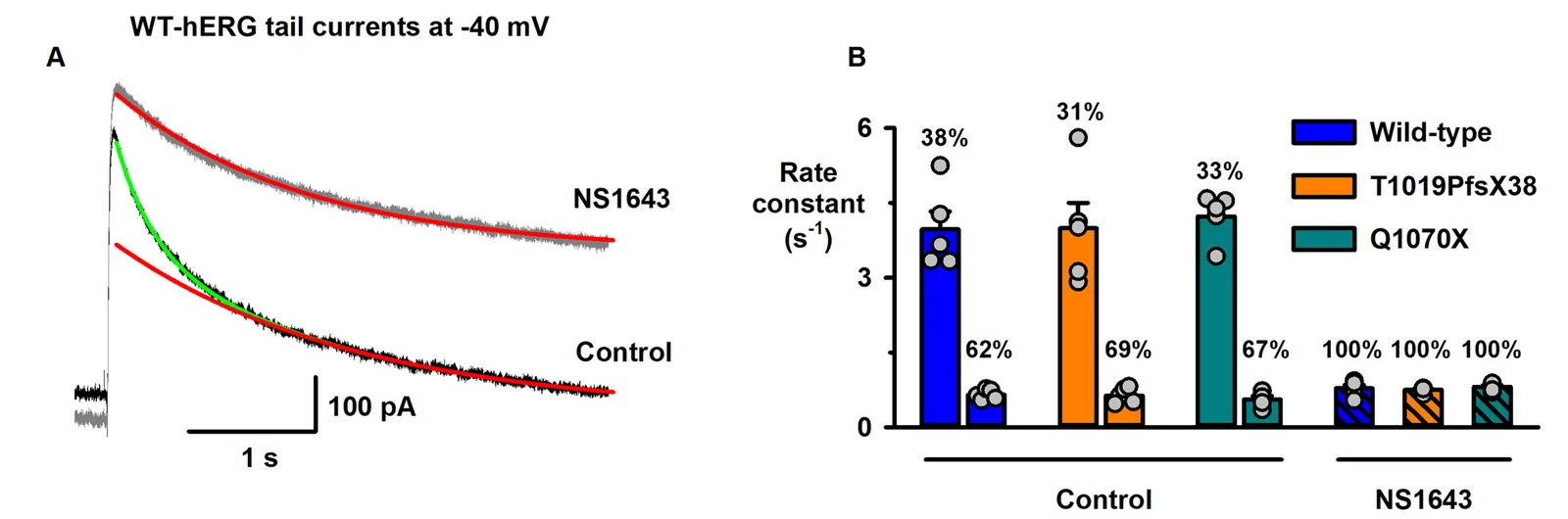
hERG channel deactivation rates in response to NS1643.** A**, Expanded tail currents recorded from WT-hERG channels before and after the addition of NS1643. WT-hERG currents were activated for 2 s at 50 mV and the tail was observed at −40 mV (as in Fig. 3A-F). Red and green lines are best 1- and 2-component exponential fits to the data. **B**, Average data of the deactivation rate constants obtained by fitting the decaying tail currents. The percentages above the bars correspond to the normalized amplitudes of the fitted exponential components. *n* = 5 each

Slow deactivation is a hallmark of hERG channel gating. The time course of tail current decay can be used to quantify the speed of deactivation at −40 mV. Inspection of recordings in Fig. 3 revealed that all tested variants deactivated slowly under control conditions, and NS1643 further slowed this process (Fig. 3D–F). Figure 6 illustrates superimposed tail currents from WT-hERG activated at 50 mV, before and after NS1643 application, showing that the drug altered tail current decay shape.

To characterize this effect, the decaying tail currents were fitted with exponential functions. The fits were assessed by visual inspection and by comparing correlation coefficients (R²) and standard deviations (SD), obtained from pClamp analysis software. Under control conditions, tail currents displayed biphasic decay (Fig. 6A), with two-component exponential fits yielding better correlation and SD compared to single-component fits (*p* < 0.01, Table S1). After NS1643 treatment, tail current decay became monophasic (Fig. 6A), and single-exponential fits were as effective as two-component fits (Table S1), consistently across all variants.

Summary data (Fig. 6B) show that, under control, the fast component accounted for 30–40% of the initial tail current. The slower component had 5–7 times longer decay time constant and accounted for 60–70% of the initial tail amplitude. NS1643 either abolished or rendered negligible the fast component, as shown in Fig. 6A and Table S1. The slow decay rates were not significantly affected by NS1643 in WT- (*p* = 0.189) and T1019PfsX38-hERG (*p* = 0.177), but were modestly accelerated in Q1070X-hERG (*p* = 0.0118). Overall, NS1643 diminished fast hERG deactivation, biasing open channels to close through the native slow-relaxation mode.

## Discussion

In this study, several key findings were identified regarding the actions of NS1643 on WT-, T1019PfsX38-, and Q1070X-hERG channels and on endogenous HEK293 currents. First, NS1643 enhanced repolarization currents in cells expressing all three hERG variants. Second, NS1643 shifted the voltage dependence of hERG activation by approximately 20 mV in the negative direction without altering maximal current amplitudes. Third, NS1643 accelerated hERG activation kinetics across a range of membrane potentials. Fourth, NS1643 altered the deactivation pattern of hERG channels by suppressing the fast deactivation component and favoring the slower relaxation mode. Finally, NS1643 inhibited endogenous, non-hERG currents in HEK293 cells. These findings are discussed below in the context of current understanding of hERG channel physiology and pharmacology.

Pharmacological enhancement of ion channel function has been successfully exploited for treating ion channel diseases such as cystic fibrosis, where small-molecule modulators restore defective CFTR activity [30]. Variant-specific functional characterization therefore provides an important framework for developing precision therapies targeting hERG dysfunction. Consistent with Bhuiyan et al. [20], Q1070X-hERG behaved similarly to WT-hERG under AP clamp conditions (Fig. 1). Moreover, in agreement with Al Salmani et al. [23], T1019PfsX38-hERG caused reduced repolarization currents (Figs. 1 and S3). NS1643 increased the repolarization currents by both disease-causing variants, but the magnitude of potentiation depended on AP duration, with larger effects for the 400 ms waveform (approximately 50–80% increase in I_integral_) than for the 1000 ms waveform (about 10–27% increase). These observations indicate that NS1643 action is time dependent and more effective when channels have not yet reached maximal activation level.

The present data demonstrate that NS1643 modulates both the voltage dependence and kinetics of hERG activation. The voltage dependence of activation reflects the fraction of channels in the open state at a given potential, whereas activation rates indicate how fast channels approach this steady state. NS1643 produced a leftward shift of approximately 20 mV in the activation curve without changing maximal tail current amplitude (Fig. 3), and increased activation rates several-fold at all tested potentials (Fig. 4). These effects are consistent with previous studies in mammalian expression systems, which reported that NS1643 facilitates hERG opening without markedly changing maximal open probability [25, 26]. Thus, NS1643 primarily lowers the energetic barrier for activation at more negative potentials rather than increasing the final fraction of open channels.

Once activated at depolarized potentials, hERG channels inactivate rapidly and remain non-conducting until repolarization, at which period they reopen rapidly then deactivate slowly to allow efficient K^+^ efflux. In this study, NS1643 did not measurably affect recovery from inactivation (data not shown), but it markedly altered deactivation kinetics. Under control conditions, tail current decay was biphasic, indicating that open channels occupy at least two kinetically distinct states with different deactivation rates (O1 and O2; Fig. 6). Approximately one-third of channels deactivated through a fast pathway (O1), while two-thirds followed a slower pathway (O2). NS1643 eliminated or greatly reduced the fast O1 component, resulting in predominantly monophasic, slow deactivation (Fig. 6, Table S1). This suggests that NS1643 alters the proportions of channels that are dwelling at different open configurations during repolarisation. An analogous effect of NS1643 on the deactivation profile of K_V_ channels was observed in studies of rat ERG2 (rERG2) and hERG1b (K_V_11.1b) but not hERG1a channels [31, 32]. These studies utilised high intracellular K^+^ [1 M] and a Xenopus oocyte expression system which might have not captured the full effect of the drug on the relative amplitudes of hERG1a channel gating components. Functionally, the observed shift toward slow deactivation is expected to prolong hERG channel availability during repolarization, potentially increasing K^+^ efflux. The precise impact on overall repolarization requires further analysis.

The combined modulation of activation and deactivation provides a plausible mechanism for the increases in repolarization I_integral_ observed under AP clamp. NS1643 was more effective at enhancing repolarization currents with short AP stimulus than with long AP stimulus (Fig. 2 vs. Figure S3). The drug is thus more effective when depolarisation is brief. For the 400 ms AP waveform, channels may not reach maximal state of activation, allowing a window for NS1643 to accelerate activation kinetics. In contrast, the 1000 ms AP waveform likely induces a near-maximal state of activation, limiting the additional benefit of faster activation in the presence of NS1643. These considerations support the view that NS1643 potentiates hERG primarily by accelerating activation and stabilizing the open state during repolarization, rather than by increasing the maximal open probability.

Finally, NS1643 inhibited endogenous currents in HEK293 cells (Fig. 2), highlighting that this compound is not selective for hERG. Similar off-target actions have been reported, including inhibition of K_V_12.1 by this compound [33]. An analogue of this drug named NS3623 blocks Cl^−^ channel while possessing hERG potentiation activity [34, 35]. Despite these limitations, NS1643 remains a valuable pharmacological probe for dissecting hERG gating mechanisms and for exploring proof-of-concept rescue strategies in LQT2 models. However, the presence of NS1643-sensitive endogenous conductances in HEK293 cells underscores the need to carefully account for non-hERG currents when interpreting functional data in this widely used expression system.

### Study limitations

This study was performed at room temperature and using mammalian cells that express drug-sensitive endogenous currents. NS1643 mainly altered the kinetics of channel activation and deactivation, which differ between room temperature and body temperature. To better understand the effect of the drug on hERG channel kinetics, additional investigations should be performed at 37 ℃. The unknown pattern of cell surface expression and the effect of NMD on T1019PfsX38- and Q1070X-hERG limits NS1643 monotherapy potential. Furthermore, the use of a relatively high NS1643 concentration (10 µM) may carry a potential risk of proarrhythmic effects. Therefore, further studies are required to determine the optimal therapeutic concentration that maximizes hERG channel enhancement while minimizing potential proarrhythmic effects.

## Conclusion

Overall, this study characterizes the effects of NS1643 on WT-, T1019PfsX38-, and Q1070X-hERG channels. hERG potentiators can rescue LQT2 variants that have residual or pharmacologically/experimentally corrected surface expression. Further work is required to assess NS1643 effectiveness under conditions that closely mimic native human physiology, including the use of patient-derived cardiomyocytes.

## Supplementary Information

Below is the link to the electronic supplementary material.

Supplementary Material 1 (PDF 1.94 MB)

## Data Availability

Data is available upon requests directed to the corresponding author: Dr. Majid Al Salmani.
