# Supplementary material for: NS1643 enhances the activity of T1019PfsX38 and Q1070X hERG channels associated with long QT syndrome type 2
Source: Pflugers Arch. 2026 Oct 2;478(10):84. doi: 10.1007/s00424-026-03215-7 (PMC13633300; doi:10.1007/s00424-026-03215-7)
Supplement: Supplementary file 1 [file 424_2026_3215_MOESM1_ESM.pdf]

Supplemental data:

			1-component exponential deactivation	2-component exponential deactivation	p value (t-test)
WT-hERG	Control (n = 5)	R^2	0.955 ± 0.004	0.979 ± 0.004	0.008
		SD	13.569 ± 3.099	5.807 ± 0.775	0.041
	NS1643 (n = 5)	R^2	0.980 ± 0.003	0.984 ± 0.004	0.367
		SD	8.543 ± 1.719	6.157 ± 0.495	0.310
T1019PfsX38-hERG	Control (n = 5)	R^2	0.960 ± 0.005	0.980 ± 0.002	0.005
		SD	19.217 ± 10.248	7.394 ± 1.891	0.222
	NS1643 (n = 5)	R^2	0.983 ± 0.002	0.985 ± 0.002	0.222
		SD	5.805 ± 0.800	5.210 ± 0.502	0.546
Q1070X-hERG	Control (n = 5)	R^2	0.967 ± 0.005	0.988 ± 0.001	0.005
		SD	16.441 ± 3.221	6.520 ± 0.571	0.016
	NS1643 (n = 5)	R^2	0.988 ± 0.001	0.992 ± 0.001	0.030
		SD	10.448 ± 0.913	6.617 ± 0.931	0.019

Table S1. Measurements of the goodness of fit for determining the likely pattern of tail current decay under control and drug treatment condition. The table reports values used for the assessment of the goodness of fit in order to decide whether one or two exponential components are necessary to reliably fit the decaying pattern of tail currents at -40 mV, as in Figure 6A. The highlighted cells are corresponded to the best values of goodness of fit. Other details are explained in the Methods and Results sections.

Figure S1:

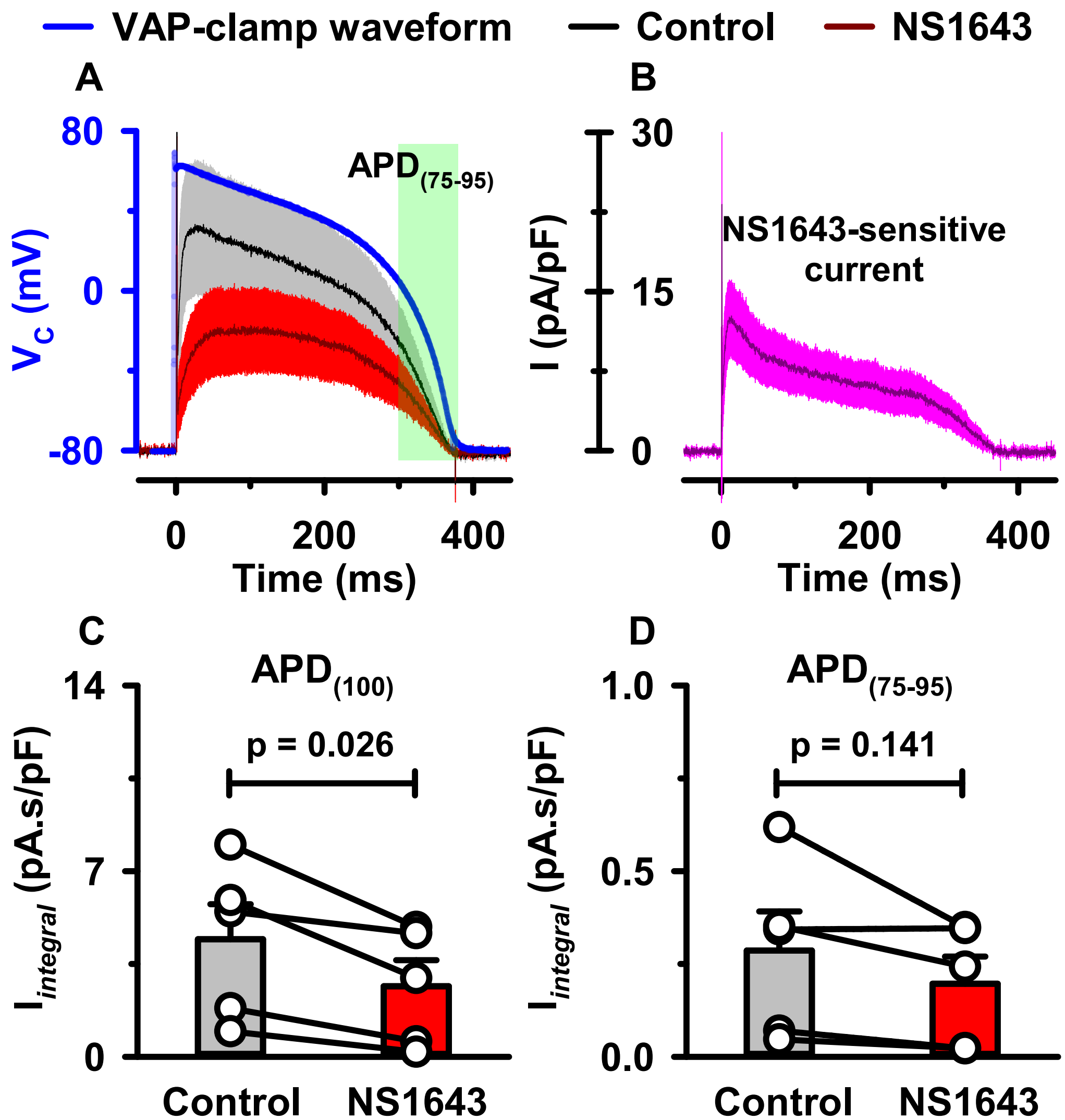

Figure S1: AP clamp measurements on untransfected HEK293 cells using a 400 ms waveform duration. A , Average whole-cell currents from HEK293 cells under control (Black / Gray) and after the addition of NS1643 [10 µM] (Red). We applied a ventricular AP clamp waveform (blue plot in A) and modified the obtained recordings using P/4 leak subtraction protocol. B, Data obtained by subtracting the recordings in A. **C-D,** Summary data of the repolarization current integrals ($I_{integral}$) measured as the area under the curve of the full AP (APD100) or the area between between 300 and 380 ms (APD75 to 95%) of the AP duration. Other details as in the Figure 1, the Methods and the Results sections.

Figure S2

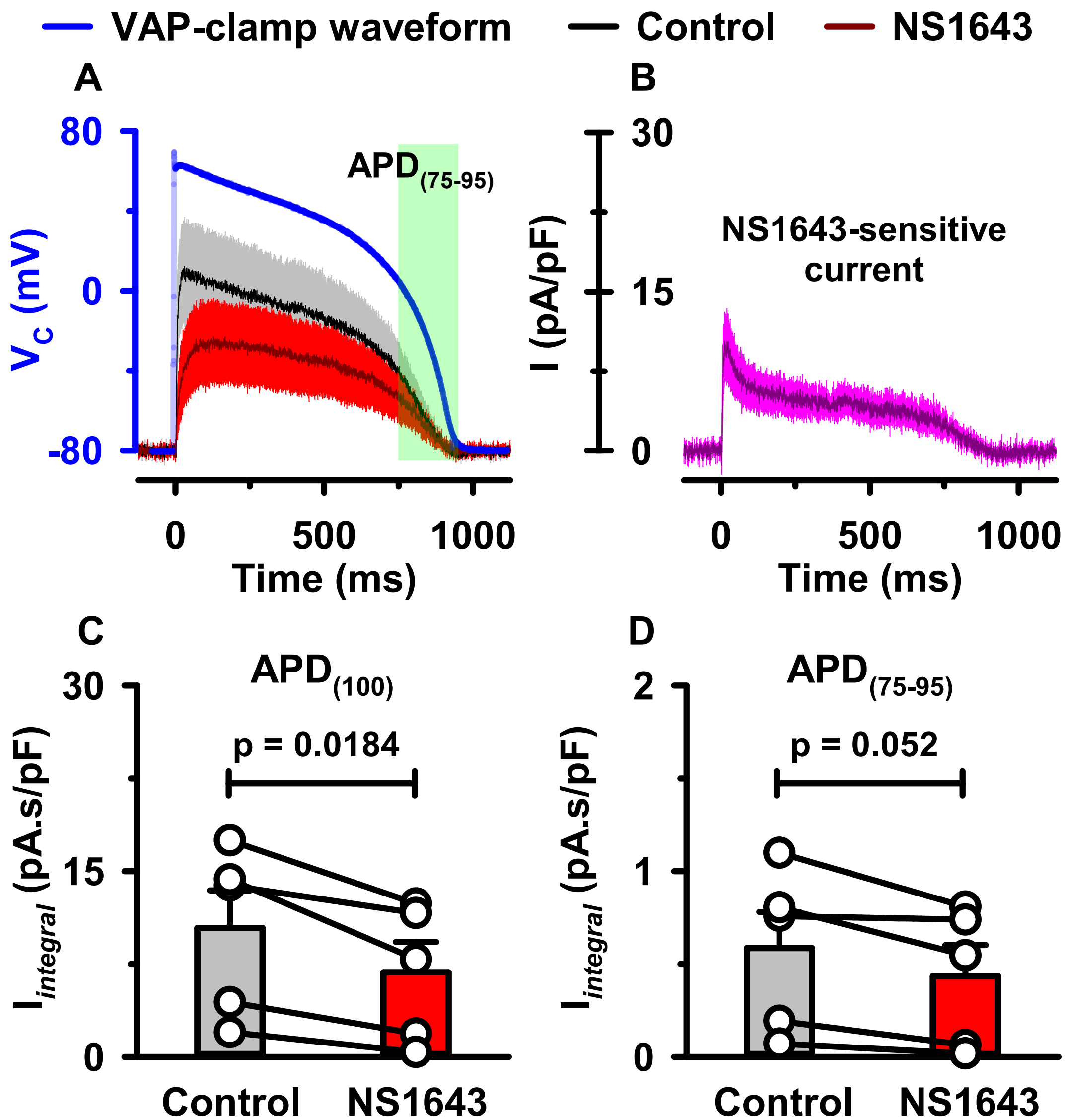

Figure S2: AP clamp measurements on untransfected HEK293 cells using a 1000 ms waveform duration. A , Average whole-cell currents from HEK293 cells under control (Black / Gray) and after the addition of NS1643 [10 µM] (Red). **B,** Data obtained by subtracting the recordings in A. **C-D,** Summary data of the repolarization current integrals ($I_{integral}$) measured as the area under the curve of the full AP (APD100) or the area between between 750 and 950 ms (APD75 to 95%) of the AP duration. Other details as in the Figure 1 and S1, and the Methods and the Results sections.

Figure S3.

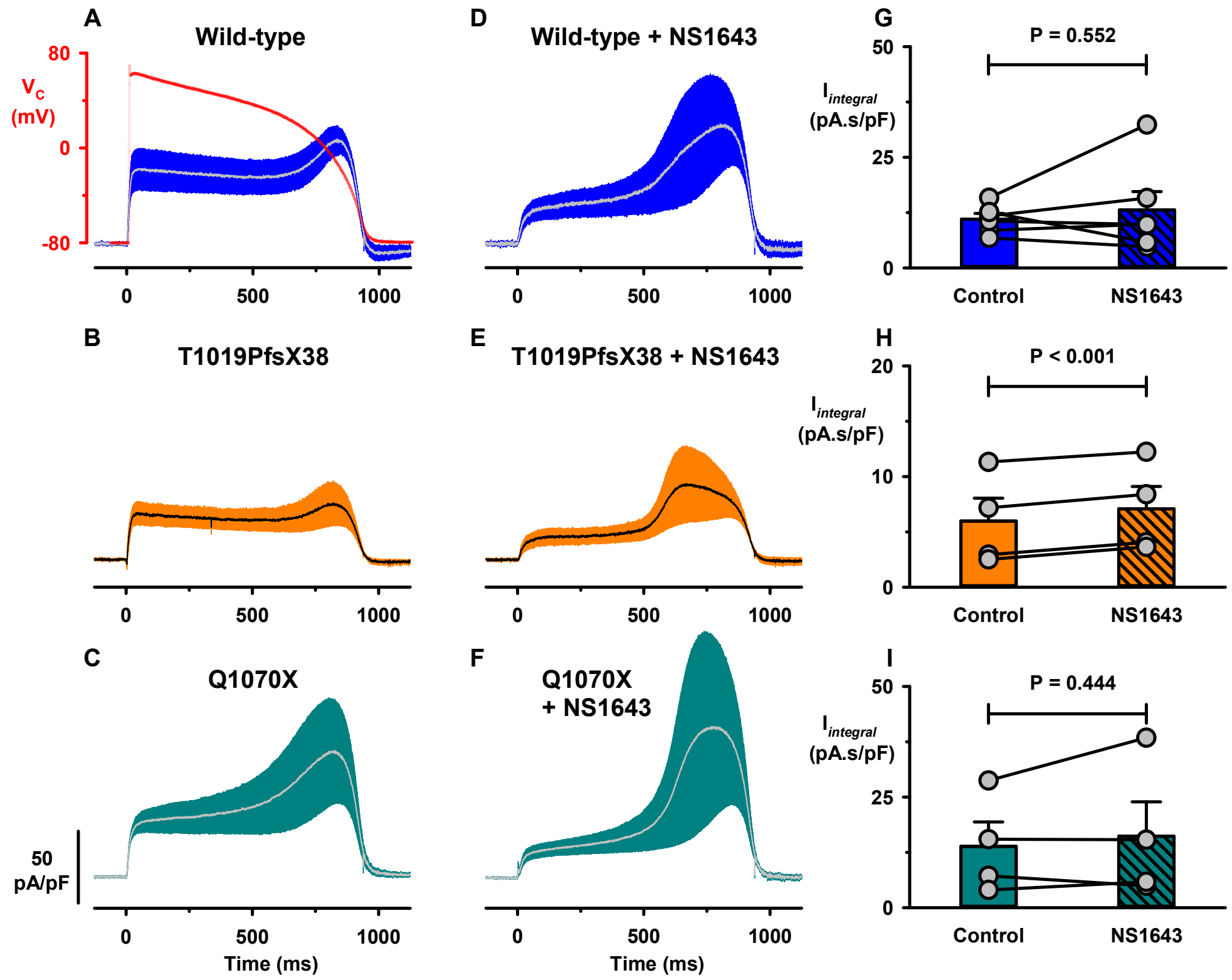

Figure S3: AP clamp measurements of the effects of NS1643 on wild-type-, T1019PfsX38- and Q1070X-hERG. A - F, Average whole-cell currents from cells expressing different channel variants under control and drug treatment conditions. We applied a ventricular AP clamp waveform (pink plot in A) and modified the obtained recordings using P/4 leak subtraction protocol. **G-I,** Summary data of the repolarization current integrals ($I_{integral}$) measured as the area under the curve between 750 and 950 ms (75 to 95%) of the AP duration. The treatment points in G - I were corrected for NS1643-induced inhibition, using the data in Figure S2. Other details as in Figure 1 and S2, and the Methods and the Results sections.
